# Balance between innate versus adaptive immune system and the risk of dementia: a population-based cohort study

**DOI:** 10.1186/s12974-019-1454-z

**Published:** 2019-03-30

**Authors:** Kimberly D. van der Willik, Lana Fani, Dimitris Rizopoulos, Silvan Licher, Jesse Fest, Sanne B. Schagen, M. Kamran Ikram, M. Arfan Ikram

**Affiliations:** 1 Department of Epidemiology, Erasmus MC - University Medical Center Rotterdam, PO Box 2040, 3000CA Rotterdam, the Netherlands; 2 Department of Psychosocial Research and Epidemiology, Netherlands Cancer Institute, Amsterdam, the Netherlands; 3 Department of Biostatistics, Erasmus MC - University Medical Center Rotterdam, Rotterdam, the Netherlands; 4 Department of Surgery, Erasmus MC - University Medical Center Rotterdam, Rotterdam, the Netherlands; 5 Department of Psychology, University of Amsterdam, Amsterdam, the Netherlands; 6 Department of Neurology, Erasmus MC - University Medical Center Rotterdam, Rotterdam, the Netherlands

**Keywords:** Innate immune system, Adaptive immune system, Dementia, Alzheimer’s disease, Epidemiology, Cohort studies

## Abstract

**Background:**

Immunity has been suggested to be important in the pathogenesis of dementia. However, the contribution of innate versus adaptive immunity in the development of dementia is not clear. In this study, we aimed to investigate (1) the association between components of innate immunity (granulocytes and platelets) and adaptive immunity (lymphocytes) with risk of dementia and (2) the association between their derived ratios (granulocyte-to-lymphocyte ratio [GLR], platelet-to-lymphocyte ratio [PLR], and systemic immune-inflammation index [SII]), reflecting the balance between innate and adaptive immunity, with risk of dementia.

**Methods:**

Blood cell counts were measured repeatedly between 2002 and 2015 in dementia-free participants of the prospective population-based Rotterdam Study. Participants were followed-up for dementia until 1 January 2016. Joint models were used to determine the association between granulocyte, platelets, and lymphocyte counts, and their derived ratios with risk of dementia.

**Results:**

Of the 8313 participants (mean [standard deviation] age 61.1 [7.4] years, 56.9% women), 664 (8.0%) developed dementia during a median follow-up of 8.6 years. Doubling of granulocyte and platelet counts tended to be associated with an increased risk of dementia (HR [95%CI] 1.22 [0.89–1.67] and 1.45 [1.07–1.95], respectively). Doubling of the derived ratios GLR, PLR, and SII were all associated with an increased dementia risk (HR [95%CI] 1.26 [1.03–1.53], 1.27 [1.05–1.53], and 1.15 [0.98–1.34], respectively).

**Conclusions:**

GLR, PLR, and SII are associated with an increased risk of dementia in the general population. This supports the role of an imbalance in the immune system towards innate immunity in the pathogenesis of dementia.

**Electronic supplementary material:**

The online version of this article (10.1186/s12974-019-1454-z) contains supplementary material, which is available to authorized users.

## Background

Dementia poses a huge burden on societies in terms of financial costs as well as on individual patients and their caregivers regarding suffering and grief [1]. Dementia is a multifactorial disease, in which various pathologies interact during the long pre-clinical phase, ultimately resulting in its clinical manifestations of cognitive decline and loss of independence. While amyloid depositions, neuronal loss, and vascular damage have long been established as key pathologies underlying dementia [2], recent findings point towards a key role for the immune system [3–5]. The immune system is a highly complex system involving multiple synergistic and antagonistic substrates, yet broadly can be classified into two components, i.e., innate immunity and adaptive immunity [6]. Innate immunity refers to immune responses present at birth, forming a first line of defense, whereas adaptive immunity is acquired during life by exposure to specific antigens [7]. High activity of innate immunity can lead to disrupted neuronal integrity and ultimately to cell death [8]. Although these components of the immune system work closely together, adaptive immunity is considered to be more neuroprotective than innate immunity, presumably by stimulating phagocytosis of amyloid fibrils [9, 10].

Exact quantification of these opposing components of the immune system is challenging and focus of ongoing research, but recent work from the field of cancer research suggests that easily obtainable laboratory measurements may in fact capture their relative activity levels to a reliable degree [11]. Measuring granulocytes, including the most abundant subtype neutrophils, and platelets provides important markers of the innate immunity, whereas measuring lymphocytes yields information on the adaptive immunity [12, 13]. Furthermore, combining these measurements into ratios, i.e., the neutrophil-to-lymphocyte ratio (NLR), platelet-to-lymphocyte ratio (PLR), and systemic immune-inflammation index (SII), is thought to even better reflect the relative balance between innate and adaptive immunity [11, 14–16]. Previous work on the link between innate versus adaptive immunity and dementia showed higher NLR and PLR in dementia patients compared to healthy individuals [17–19]. Yet, to really understand the role of the immune system in the risk of developing dementia, it is pivotal to study how these markers change during the pre-clinical phase of the disease.

We thus investigated the longitudinal association of markers of the innate versus adaptive immune system with the risk of dementia. The underlying hypothesis was that higher activity of the innate versus adaptive immune system would be associated with an increased risk of dementia. A further methodological novelty of our study was the use of joint modeling that enabled us to study the longitudinal evolution of the various markers during the pre-clinical phase in conjunction with survival analyses.

## Methods

### Study population

The present study is embedded in the Rotterdam Study, a prospective population-based cohort study in Rotterdam, the Netherlands. The Rotterdam Study started in 1990 with 7983 persons (response of 78%) aged ≥ 55 years and residing in the district Ommoord, a suburb of Rotterdam. This first subcohort (RS-I) was extended with a second subcohort (RS-II) in 2000, consisting of 3011 persons (response of 67%), and with a third subcohort (RS-III) in 2006, composed of 3932 persons aged ≥ 45 years (response of 65%). The design of the Rotterdam Study has been described in detail previously [20]. In brief, participants were examined in detail at study entry and at follow-up visits every 3 to 5 years. They were interviewed at home by a trained research nurse, followed by two visits at the research facility for additional interviewing, laboratory assessments, imaging, and physical examinations.

The Rotterdam Study was approved by the Medical Ethics Committee of Erasmus Medical Center and by the board of The Netherlands Ministry of Health, Welfare, and Sports. A written informed consent was obtained from all participants.

**Fig. 1:**
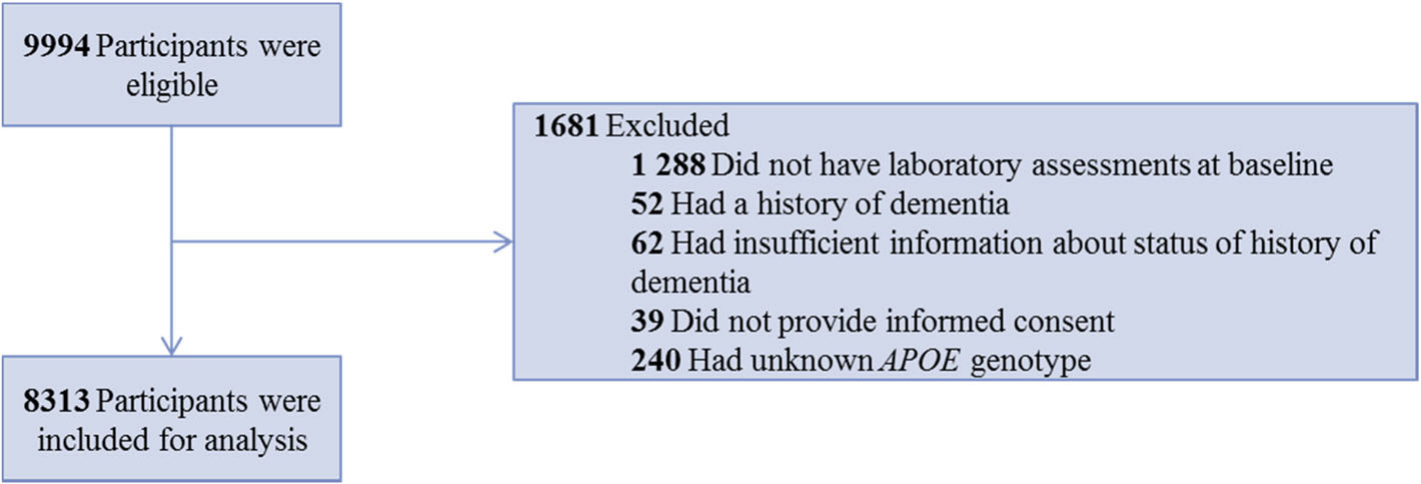
Flowchart participants for analysis association between blood cell counts, and their derived ratios, and dementia. Abbreviations: *APOE*, apolipoprotein E

Laboratory tests for granulocytes, platelets, and lymphocytes were introduced from 2002 onwards, corresponding with the following assessment rounds in the Rotterdam Study (baseline in this study): i.e., fourth round of RS-I, second round of RS-II, and first round of RS-III, comprising 9994 participants. From these 9994 eligible participants, we excluded those without complete baseline blood measurements (*n* = 1288). Of the remaining participants, we excluded those with a history of dementia (*n* = 52), participants who were insufficiently screened for dementia (*n* = 62), and those without informed consent to assess medical records during follow-up (*n* = 39). Lastly, we excluded participants with missing apolipoprotein E (*APOE*) genotype (*n* = 240), resulting in 8313 participants for analysis (flowchart in Fig. 1).

### Assessment of blood cell counts and their derived ratios

Fasting blood samples were taken during each visit at the research center with a maximum of three visits during follow-up. Full blood count measurements were performed using the COULTER® Ac·T diff2™ Hematology Analyzer (Beckman Coulter, San Diego, CA, USA) directly after blood sample drawn. Laboratory measurements included absolute granulocyte, platelet, and lymphocyte counts in 10^9^ per liter.

Since neutrophil counts were not available, we used granulocyte count as a reliable proxy given that these are the most abundant subtype of neutrophils [21, 22].

The granulocyte-to-lymphocyte ratio (GLR) and PLR were calculated as the ratio of granulocyte count to lymphocyte count and as the ratio of platelet count to lymphocyte count, respectively. The SII was defined as platelet count times the GLR.

### Assessment of dementia

Participants were screened for dementia at baseline and subsequent center visits with the Mini-Mental State Examination and the Geriatric Mental Schedule organic level [23]. Those with a Mini-Mental State Examination score < 26 or Geriatric Mental Schedule score > 0 underwent further investigation and informant interview, including the Cambridge Examination for Mental Disorders of the Elderly. The entire cohort was continuously under surveillance for dementia through electronic linkage of the study database with medical records from general practitioners and the regional institute for outpatient mental health care. Available information on clinical neuroimaging was used when required for diagnosis of dementia subtype. A consensus panel led by a consultant neurologist established the final diagnosis according to standard criteria for dementia (Diagnostic and Statistical Manual of Mental Disorders III-revised), Alzheimer’s disease (AD; National Institute of Neurological and Communicative Disorders and Stroke and the Alzheimer’s Disease and Related Disorders Association), and vascular dementia (National Institute of Neurological Disorders and Stroke and Association Internationale pour la Recherché et l’Enseignement en Neurosciences). Follow-up until 1 January 2016 was virtually complete (93.8% of potential person-years observed).

### Other measurements

We assessed education and smoking by interview. Education level was classified into primary education, lower (lower general education, intermediate general education, or lower vocational education), intermediate (intermediate vocational education or higher general education), or higher (higher vocational education or university). Smoking status was categorized as never, former, or current smoker. Body mass index (BMI) was computed from measurements of height and weight (kg/m^2^). Diabetes mellitus was defined as use of antidiabetic medication, fasting serum glucose level ≥ 7.1 mmol/L, or random serum glucose level ≥ 11.1 mmol/L [24]. History of stroke was assessed by interview and verified by reviewing medical records [25]. *APOE* genotype was determined using polymerase chain reaction on coded DNA samples in RS-I and with a bi-allelic TaqMan assay in the two extensions (RS-II and RS-III) [26, 27]. *APOE* ε4 carrier status was defined as carrier of one or two *APOE* ε4 alleles.

### Statistical analysis

We associated the different blood cell counts and their derived ratios with the risk of all-cause dementia using the framework of joint models for longitudinal and survival data. In this way, we are able to account for the endogenous nature (i.e., blood cell counts can be measured with error during follow-up and their values at any time point can be affected by an event occurring at an earlier time point) [28] and the correlations in the repeated measurements of granulocyte, platelet, and lymphocyte counts [29].

In order to normalize the skewed distribution of granulocyte, platelet, and lymphocyte counts, and their derived ratios, we used a natural logarithmic transformation. Hazard ratios (HRs) with 95% confidence intervals (CIs) were obtained from the joint models, using the piecewise-constant baseline hazard, and multiplied with log(2), providing a HR for doubling of the blood cell counts and their ratios. We computed two nested models: model I was adjusted for baseline age (continuous, centered as age minus mean age) and sex; model II was additionally adjusted for education, smoking status, BMI (continuous), diabetes mellitus, history of stroke, and *APOE* ε4 carrier status. For assessment of the association between the individual components of the ratios and dementia, we repeated analyses with adjustment for the baseline blood cell counts of the remaining two blood cell types (for instance, the association of granulocyte count with dementia was adjusted for platelet and lymphocyte counts). Follow-up time was used as timescale and started at the first laboratory assessment until the date of all-cause dementia diagnosis, death, loss to follow-up, or 1 January 2016, whichever came first. Censoring participants at date of death allowed us to compute cause-specific HRs.

In sensitivity analyses, we repeated all analyses using age as timescale instead of follow-up time to account for potential residual confounding by age and to minimize potential effects of left truncation. We additionally censored for stroke events during follow-up to preclude that the observed effect may be driven by incident strokes that occurred before dementia diagnosis. Moreover, we investigated the association between the ratios and AD or vascular dementia separately. Lastly, we explored effect modification by stratifying by median age, sex, smoking status, diabetes mellitus, and *APOE* ε4 carrier status.

Multiple imputation was used for missing covariates (maximum of 0.99%), with five imputed datasets based on other covariates and the outcome. Rubin’s method was used for pooled HRs and 95% Cis [30]. Two-sided *P* < .05 was considered statistically significant. Statistical analyses were performed using the R packages “survival”, “nlme”, “JM”, and “JMbayes” in RStudio Version 3.3.2 [28, 29, 31, 32].

## Results

Characteristics of included and excluded study participants are presented in Table 1. An overview of the median blood cell counts and blood cell-based ratios per assessment round is shown in Additional file 1: Table S1. Mean age of included study participants was 61.1 years and 56.9% were women. During a median follow-up of 8.6 years (70,273 person-years), 664 participants developed all-cause dementia (543 AD, 31 vascular dementia) with an incidence rate of 9.4 (95% CI, 8.7–10.2) per 1000 person-years.

**Table 1:** Baseline characteristics of the included and excluded study participants

Characteristic	Included participants (*N* = 8313)	Excluded participants (*N* = 1528)^#^	
		No blood measurements (*N* = 1288)	Unknown *APOE* genotype (*N* = 240)
Age, year, mean (SD)	61.1 (7.4)	72.6 (11.8)	61.7 (8.2)
Women	4729 (56.9)	845 (65.6)	160 (66.7)
Education			
Primary	908 (11.0)	233 (18.4)	25 (11.7)
Lower	3329 (40.3)	537 (42.4)	95 (44.4)
Intermediate	2429 (29.4)	336 (26.5)	57 (26.7)
Higher	1588 (19.2)	161 (12.7)	37 (17.3)
Body mass index, kg/m^2^, mean (SD)	27.6 (4.3)	27.6 (4.5)	28.2 (4.8)
Smoking status			
Current	1595 (19.3)	308 (24.4)	57 (24.5)
Former	4191 (50.7)	550 (43.6)	106 (45.5)
Diabetes mellitus	501 (6.0)	136 (10.7)	15 (6.3)
History of stroke	305 (3.7)	54 (4.2)	11 (4.6)
*APOE* ε4 carrier status	2328 (28.0)	244 (30.8)	
Blood cell types, 10^9^/L, median (IQR)			
Granulocytes	3.8 (1.6)		4.0 (1.7)
Platelets	263 (84)		277 (87)
Lymphocytes	2.2 (0.8)		2.3 (0.9)
Blood cell-based ratios, median (IQR)			
Granulocyte-to-lymphocyte ratio	1.7 (0.9)		1.7 (0.8)
Platelet-to-lymphocyte ratio	120 (55)		119 (54)
Systemic immune-inflammation index	455 (280)		473 (312)

*Abbreviations*: *APOE* apolipoprotein E, *IQR* interquartile ratio, *N* number of participants, *SD* standard deviation

Values are shown before multiple imputation and therefore not always add up to 100%

Data are presented as number (percentage) of participants unless otherwise indicated

^#^Excluded participants in this table only include those participants who were excluded due to no complete blood measurements or unknown *APOE* ε4 carrier status

Higher levels of granulocytes reflecting higher innate immunity were associated with an increased risk of dementia, but only after correcting for the platelet and lymphocyte counts (HR for doubling granulocyte count [95% CI] = 1.33 [0.99–1.79], Table 2). Doubling of platelets was associated with an increased risk of dementia (HR [95% CI] = 1.48 [1.11–1.96]). Regarding adaptive immunity, higher levels of lymphocytes were associated with a decreased risk of dementia (HR for doubling lymphocyte count [95% CI] = 0.80 [0.64–0.99]).

**Table 2:** Association between blood cell counts and derived ratios, and risk of all-cause dementia

Laboratory assessment^#^	All-cause dementia (*n*/*N* = 664/8313)	
	Model I	Model II
	HR (95% CI)	HR (95% CI)
Granulocytes	1.14 (0.87–1.50)	1.07 (0.80–1.43)
Corrected for platelets and lymphocytes	1.33 (0.99–1.79)	1.22 (0.89–1.67)
Platelets	1.48 (1.11–1.96)^*^	1.43 (1.08–1.90)^*^
Corrected for granulocytes and lymphocytes	1.48 (1.10–2.00)^*^	1.45 (1.07–1.95)^*^
Lymphocytes	0.80 (0.64–0.99)^*^	0.81 (0.64–1.03)
Corrected for granulocytes and platelets	0.76 (0.61–0.96)^*^	0.78 (0.62–1.00)
Granulocyte-to-lymphocyte ratio	1.34 (1.10–1.63)^*^	1.26 (1.03–1.53)^*^
Platelet-to-lymphocyte ratio	1.29 (1.08–1.55)^*^	1.27 (1.05–1.53)^*^
Systemic immune-inflammation index	1.18 (1.02–1.39)^*^	1.15 (0.98–1.34)

*Abbreviations*: *CI* confidence interval, *HR* hazard ratio, *n* number of incident dementia events, *N* number of participants for analysis. Model I is adjusted for age and sex. Model II is adjusted for age, sex, education, smoking status, body mass index, diabetes mellitus, history of stroke, and *APOE4* ε4 carrier status

^#^All types of blood cells and their derived ratios were natural logarithmic transformed

^*^Indicates statistically significant result

Higher levels of GLR, PLR, and SII were associated with an increased dementia risk (HR [95% CI] for doubling GLR = 1.34 [1.10–1.63]; for PLR = 1.29 [1.08–1.55]; for SII = 1.18 [1.02–1.39], respectively (Table 2)). Risk estimates were comparable when using the adjusted model and when using age as timescale instead of follow-up time.

Censoring for stroke did not meaningfully change the risk estimates (Table 3). Higher levels of platelets showed a slightly stronger association with AD compared with all-cause dementia, while the association with granulocytes was less pronounced for AD. Risk estimates for all-cause dementia and AD were comparable for the ratios. For vascular dementia, risk estimates regarding the individual blood cell components and their derived ratios were more pronounced than for all-cause dementia, but small numbers led to wider confidence intervals (*n* = 31).

**Table 3:** Association between blood cell counts derived ratios, and risk of all-cause dementia and dementia subtypes

Laboratory assessment^#^	All-cause dementia, censored for stroke (*n*/*N* = 579/8008)^†^	Alzheimer’s disease (*n*/*N* = 543/8313)	Vascular dementia (*n*/*N* = 31/8313)
	HR (95% CI)	HR (95% CI)	HR (95% CI)
Granulocytes	1.13 (0.83–1.56)	1.03 (0.75–1.42)	1.99 (0.52–7.55)
Corrected for platelets and lymphocytes	1.36 (0.96–1.93)	1.12 (0.79–1.58)	1.92 (0.44–8.41)
Platelets	1.45 (1.07–1.96)*	1.59 (1.17–2.17)*	3.86 (1.02–14.6)*
Corrected for granulocytes and lymphocytes	1.47 (1.07–2.02)*	1.63 (1.18–2.27)*	3.39 (0.84–13.7)
Lymphocytes	0.80 (0.62–1.02)	0.85 (0.66–1.10)	0.76 (0.25–2.30)
Corrected for granulocytes and platelets	0.76 (0.58–0.98)*	0.81 (0.62–1.06)	0.64 (0.20–2.03)
Granulocyte-to-lymphocyte ratio	1.33 (1.07–1.65)*	1.17 (0.95–1.46)	1.85 (0.74–4.62)
Platelet-to-lymphocyte ratio	1.31 (1.07–1.60)*	1.30 (1.06–1.60)*	1.99 (0.82–4.81)
Systemic immune-inflammation index	1.19 (1.01–1.41)*	1.15 (0.97–1.37)	1.77 (0.87–3.63)

*Abbreviations*: *CI* confidence interval, *HR* hazard ratio, *n* number of incident dementia events, *N* number of participants for analysis. Models are adjusted for age, sex, education, smoking status, body mass index, diabetes mellitus, history of stroke, and *APOE4* ε4 carrier status

^#^All types of blood cells and their derived ratios were natural logarithmic transformed

^†^Number of participants for analysis is 8313 minus participants with a history of stroke (*n* = 305)

*Indicates statistically significant result

**Fig. 2:**
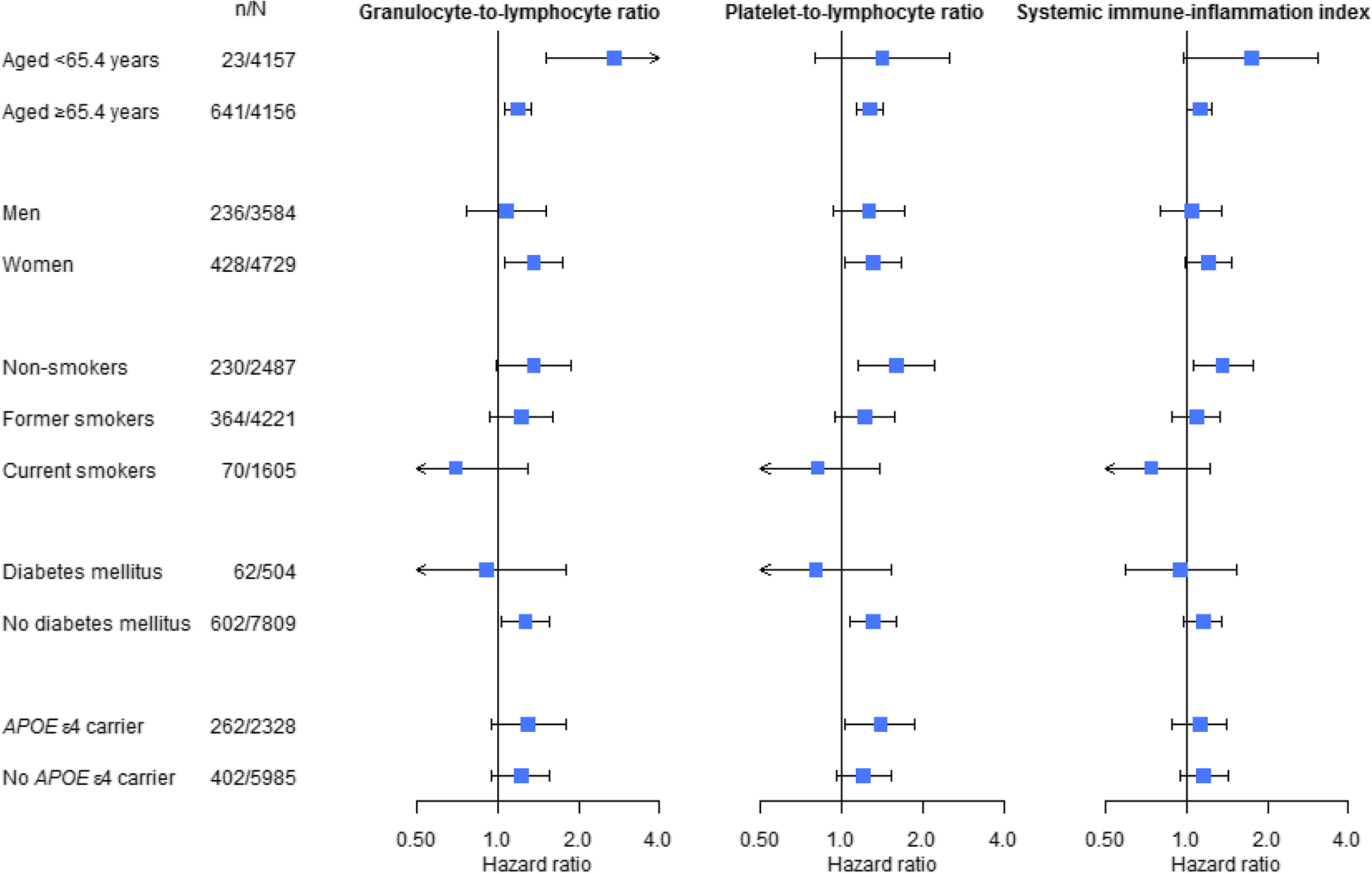
Forest plots of the association of the GLR, PLR, and SII, and risk of dementia. Hazard ratios are shown in logarithmic scale with stratification by median age, sex, smoking status, diabetes mellitus, and *APOE* ε4 carrier status. Abbreviations: *APOE*, apolipoprotein E; *GLR*, granulocyte-to-lymphocyte ratio; *n*, number of incident dementia events; *N*, number of participants for analysis; *PLR*, platelet-to-lymphocyte ratio; *SII*, systemic immune-inflammation index

**Fig. 3:**
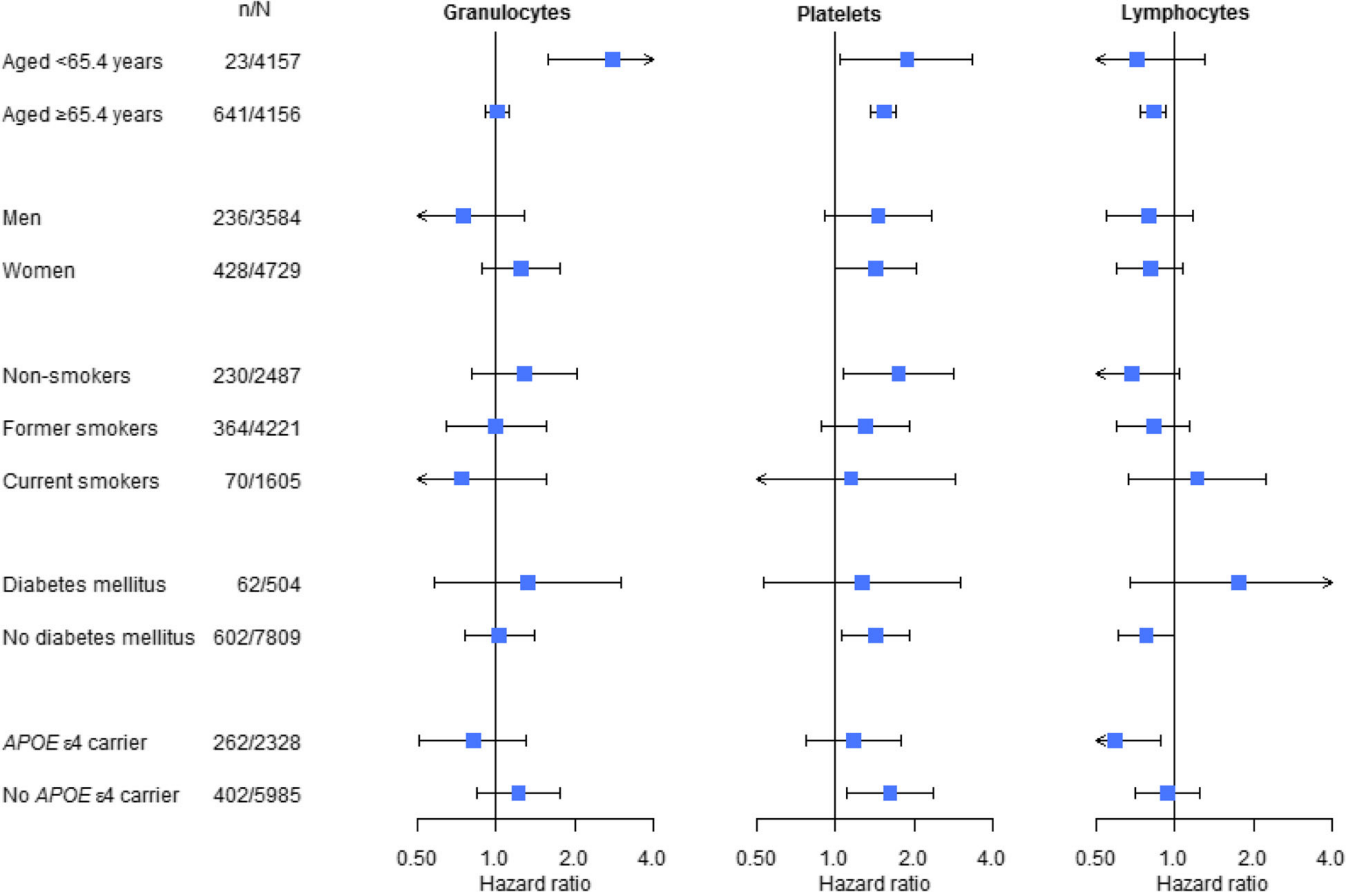
Forest plots of the association of granulocytes, platelets, and lymphocytes, and the risk of dementia. Hazard ratios are shown in logarithmic scale with stratification by median age, sex, smoking status, diabetes mellitus, and *APOE* ε4 carrier status. Abbreviations: *APOE*, apolipoprotein E; *n*, number of incident dementia events; *N*, number of participants for analysis

Stratified analyses showed that the association between the ratios and dementia was particularly pronounced in participants aged below the median age of 65.4 years, women, and non-smokers (Fig. 2). However, formal interaction terms did not reach statistical significance. Also, no significant effect modification was observed across different strata of these variables for the association between granulocyte, platelet, and lymphocyte counts, and risk of dementia (Fig. 3).

## Discussion

In this population-based study, we found that higher levels of granulocyte and platelet counts are related to an increased risk of dementia, whereas a higher lymphocyte count is associated with a decreased dementia risk. Furthermore, higher levels of their derived ratios, i.e., GLR, PLR, and SII, are associated with an increased risk of all-cause dementia, including its subtype AD and even more with vascular dementia.

Activation of the immune systems can result in inflammation by production of different cytokines [33]. These cytokines can act as a link between the innate and the adaptive immune system, having pro- or anti-inflammatory effects depending on the type of cytokine [34]. A recent meta-analysis of 175 studies suggests that AD is accompanied by an inflammatory response and that this can be reflected by a variety of systemic cytokines, for instance interferon-γ, interleukin (IL)-2, and in particular IL-6, of which dysregulation has been associated with multiple chronic inflammatory diseases [35, 36]. It is now recognized that systemic inflammation can trigger or exacerbate the inflammatory environment of the brain, thereby contributing to chronic neuroinflammation and neurodegeneration [37]. A plausible explanation for the occurrence of this chronic neuroinflammation in (pre)demented individuals involves a disruption of a process called resolution [38]. Resolution is an active process that halts the acute phase of inflammation and restores tissue homeostasis. The acute inflammatory phase is usually initiated in response to infection, neoplasia, tissue injury, or other major homeostatic stressors. This phase is accompanied by the increased release of pro-inflammatory mediators such as prostaglandins, leading to leukocyte recruitment. Normally, resolution would clear the recruited granulocytes [39]. However, it has been shown that failure of resolution, induced by any chronic inflammatory state, is associated with an overactive innate immune system, resulting in the development of chronic inflammation, which could subsequently lead to AD [38, 40, 41]. Our finding that an increase in the granulocyte count, resulting in a higher GLR and SII, is associated with an increased risk of dementia could therefore support the role of insufficient resolution in the pathogenesis of dementia.

Only few studies examined the interplay between the innate and adaptive immunity by studying levels of these blood cell-based ratios in dementia patients. Two cross-sectional studies showed that NLR and PLR were elevated in AD patients compared to dementia-free controls [17, 18]. In contrast, a longitudinal study assessing the trajectory of NLR found no significant difference in its longitudinal evolution between AD patients and dementia-free participants [19]. Although they examined differences between AD patients and dementia-free controls, they did not investigate the risk of developing dementia in dementia-free participants in relation to their levels of NLR. In the present study, we did take the time until dementia into account by a joint modeling approach and were therefore able to assess the risk of dementia in relation to the change of blood cell counts and their derived ratios.

Interestingly, recent evidence shows that the NLR and PLR are partly genetically determined with 36% estimated heritability for NLR and 64% for PLR in a healthy population [42]. Moreover, different single-nucleotide polymorphisms (SNPs) identified through genome-wide association study (GWAS) were significantly related to the PLR phenotype, but not to NLR [43]. Importantly, some but not all of these SNPs were also related to platelet, indicating that these SNPs capture the interplay between platelets and lymphocytes. Thus far, no GWAS for SII has been performed. Exploring the dementia risk by genetically predicted blood cell-based ratios may provide more insight in the causal role of immunity in dementia.

Strengths of our study include the population-based setting and the thorough follow-up for dementia. Another strength is the prospective design of this study, with the blood cell counts being measured at multiple time points. Using an innovative statistical method, we combined these repeated measurements with dementia as survival outcome. Moreover, we used blood cell counts and their derived ratios, which are low-cost and easy to implement in the clinic and other research settings. Although these ratios are proven to be associated with chronic systemic inflammation, we need to emphasize that it is unknown whether higher levels of GLR, PLR, and SII are functional and cause higher levels of pro-inflammatory cytokines. To identify the actual involved immune cell populations, determination of different cytokines is still needed. Furthermore, the innate and adaptive immune systems are overlapping, making it difficult to completely distinguish their separate effects. In addition, we used the granulocyte count as proxy for the neutrophil count. Although the relative proportion of neutrophils compared to eosinophils and basophils may be lower in persons with several specific diseases such as parasitic infections, asthma, or immune diseases, neutrophils are generally the most important subtype of granulocytes. If anything, misclassification of the granulocytes would be non-differential and would therefore lead to underestimation of the estimates [11]. In addition, we cannot rule out reversed causality, i.e., that dementia is subclinical at time of the laboratory assessments and causes higher levels of GLR, PLR, and SII. Lastly, we did not have the power to study other neurodegenerative diseases beyond dementia, such as Parkinson’s disease or amyotrophic lateral sclerosis. It would be interesting for future studies to also investigate the relation between inflammation and these diseases.

## Conclusions

In conclusion, higher levels of the ratios GLR, PLR, and SII are associated with an increased risk of developing dementia in the general population. Higher activation of the innate immune system reflected by higher levels of granulocytes and platelets is associated with an increased dementia risk, while the adaptive immune system is suggested to be more neuroprotective. These findings support the role of dysregulation of the immune systems in the pathogenesis of dementia. Further studies are warranted to assess during which phase of the pathogenesis of dementia immunity is involved and to assess causality in order to develop prevention and therapeutic strategies.

## Additional file

Additional file 1: **Table S1.** Overview of median blood cell counts and blood cell-based ratios measured per Rotterdam Study assessment round. (DOCX 18 kb)

## Abbreviations

AD: Alzheimer’s disease
*APOE*: Apolipoprotein E
BMI: Body mass index
CI: Confidence interval
GLR: Granulocyte-to-lymphocyte ratio
GWAS: Genome-wide association study
HR: Hazard ratio
IL: Interleukin
IQR: Interquartile ratio
NLR: Neutrophil-to-lymphocyte ratio
PLR: Platelet-to-lymphocyte ratio
RS: Rotterdam Study
SD: Standard deviation
SII: Systemic immune-inflammation index
SNP: Single-nucleotide polymorphisms
