# Supplementary material for: Balance between innate versus adaptive immune system and the risk of dementia: a population-based cohort study
Source: J Neuroinflammation. 2019 Mar 30;16:68. doi: 10.1186/s12974-019-1454-z (PMC6441146; doi:10.1186/s12974-019-1454-z)
Supplement: Supplementary file 1 — Table S1. Overview of median blood cell counts and blood cell-based ratios measured per Rotterdam Study assessment round. (DOCX 18 kb) [file 12974_2019_1454_MOESM1_ESM.docx]

SUPPLEMENTARY MATERIAL

**Table S1** Overview of median blood cell counts and blood cell-based ratios measured per Rotterdam Study assessment round.

| **Laboratory assessment** | **First assessment round**^#^  **(N=8313)** | **Second assessment round^†^**  **(N=5663)** | **Third assessment round^*^**  **(N=1886)** |
| --- | --- | --- | --- |
| Blood cell types, 10^9^/L, median (IQR) |  |  |  |
| Granulocytes | 3.8 (1.6) | 4.0 (1.6) | 3.6 (1.5) |
| Platelets | 263 (84) | 262 (83) | 224 (75) |
| Lymphocytes | 2.2 (0.8) | 2.2 (0.8) | 1.9 (0.9) |
| Blood cell-based ratios, median (IQR) |  |  |  |
| Granulocyte-to-lymphocyte ratio | 1.7 (0.9) | 1.8 (0.9) | 1.9 (1.1) |
| Platelet-to-lymphocyte ratio | 120 (55) | 116 (53) | 117 (59.1) |
| Systemic immune-inflammation index | 455 (280) | 461 (283) | 421 (290) |

Abbreviations: IQR, interquartile ratio; N = number of participants; RS = Rotterdam Study.

^#^ The first measurement corresponds with the fourth round of RS-I, second round of RS-II, and first round of RS-III.

^†^ The second measurement corresponds with the fifth round of RS-I, third round of RS-II, and second round of RS-III.

^*^ The third measurement corresponds with the sixth round of RS-I and the fourth round of RS-II.
